# Child maltreatment during the COVID-19 pandemic: implications for child and adolescent mental health

**DOI:** 10.3389/frcha.2024.1415497

**Published:** 2024-07-03

**Authors:** Hannah McDowell, Sophie Barriault, Tracie O. Afifi, Elisa Romano, Nicole Racine

**Affiliations:** ^1^ School of Psychology, University of Ottawa, Ottawa, ON, Canada; ^2^ Departments of Community Health Sciences and Psychiatry, Max Rady College of Medicine, University of Manitoba, Winnipeg, MB, Canada; ^3^ Children’s Hospital of Eastern Ontario Research Institute, Ottawa, ON, Canada

**Keywords:** maltreatment, COVID-19, child, adolescent, mental health

## Abstract

As societies worldwide addressed the numerous challenges associated with the COVID-19 pandemic, a troubling concern emerged—the possible rise of child maltreatment, which is a pernicious risk factor for child and adolescent mental health difficulties. This narrative review aims to provide a comprehensive understanding of how the many changes and challenges associated with the pandemic influenced worldwide occurrences of child maltreatment and, subsequently, the mental health of children and adolescents. First, we present the well-established evidence regarding the impact of child maltreatment on the mental health of children and adolescents both before and during the COVID-19 pandemic. Next, we examine the existing literature on the prevalence of child maltreatment during the pandemic, explanations for conflicting findings, and key mechanisms influencing the prevalence of maltreatment. Using a heuristic model of child maltreatment and its downstream influence on child mental health, we discuss risk and protective factors for maltreatment as well as mechanisms by which maltreatment operates to influence child and adolescent mental health. Finally, based on the accumulated evidence, we provide important recommendations for advancing research on child maltreatment, emphasizing the necessity for routine monitoring of maltreatment exposure at a population level, and discussing the implications for the field of child protection. This comprehensive review aims to contribute to the understanding of the challenges arising from the intersection of the COVID-19 pandemic and child maltreatment, with the goal of informing effective interventions in the domain of child welfare.

## 1 Introduction

The COVID-19 pandemic has profoundly impacted various elements of global society, including economies, education, health care, and government policies. In addition to these system impacts, the pandemic has significantly affected mental health. According to the World Health Organization (1), in the first year following the onset of the pandemic, diagnoses of anxiety and depression among adults increased by 25%. While the WHO has highlighted the impact the pandemic has had on global mental health, it is crucial to note that specific populations faced an elevated risk of experiencing mental health challenges, particularly children and adolescents (2). Moreover, children and adolescents from at-risk families—characterized by low socioeconomic status, living in one-parent households, having a parent with mental health disorder or substance use challenges, or a history of maltreatment—were found to be at an even greater risk for the adverse mental health effects of the pandemic. Research indicates that children and adolescents from at-risk families had significantly higher depression and anxiety scores both before and during the pandemic compared to their peers without these risk factors (3). Notably, many studies have identified a notable deterioration in children and adolescents' mental health during the COVID-19 pandemic (3–6). When considering the increase in mental health difficulties since the pandemic, it is crucial to exercise caution, as such increases may not solely stem from the pandemic. Instead, the pandemic might have magnified existing trends among children and adolescents (7). Overall, research examining the impact of the COVID-19 pandemic on mental health declines is complex and multifaceted, with several risk factors likely at play.

**Figure 1:**
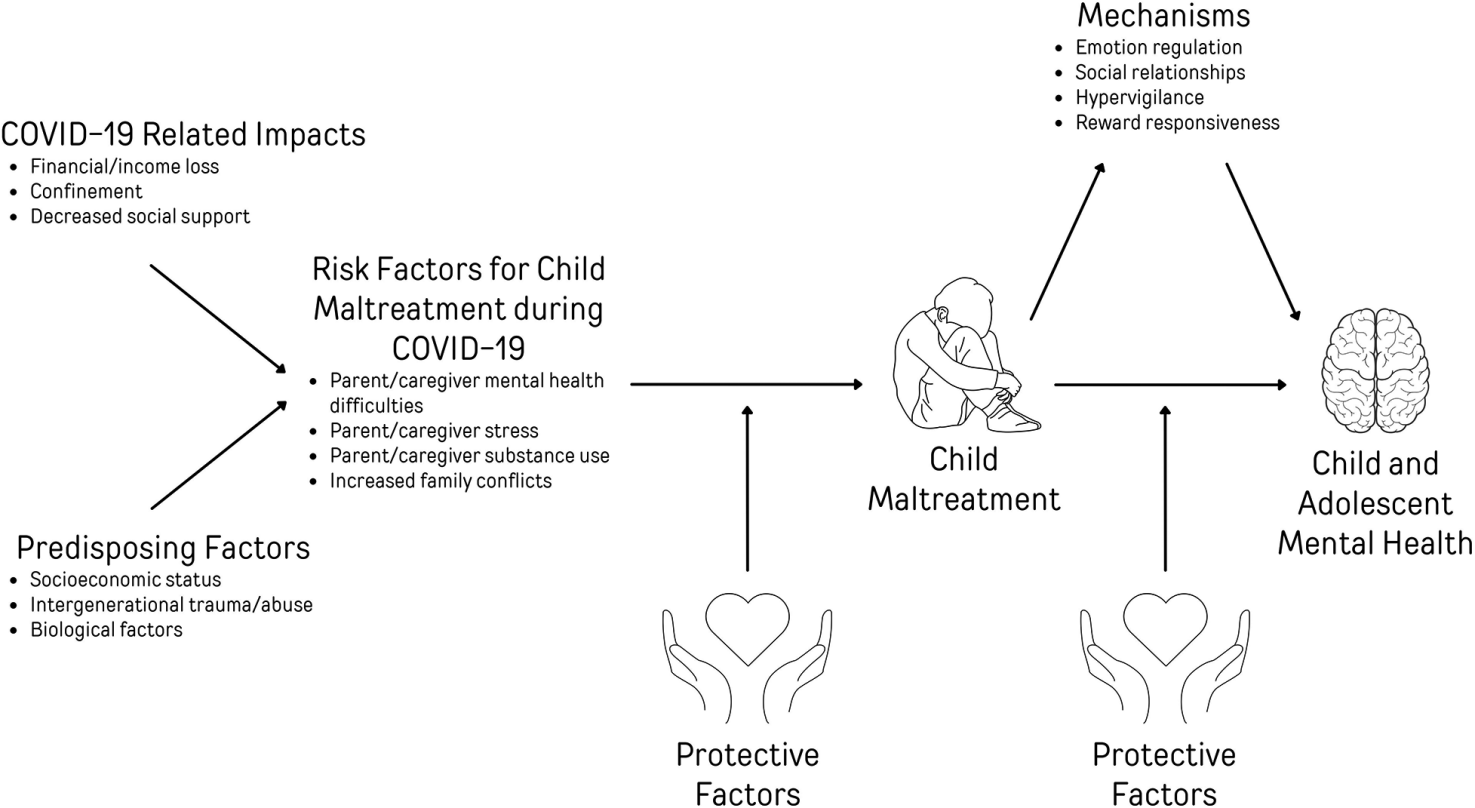
Heuristic model of risk factors for child maltreatment and child and adolescent mental health during COVID-19.

Exposure to child maltreatment, including abuse and neglect, is a robust risk factor for poor mental health among children and adolescents (8). Conditions for an uptick in child maltreatment during the COVID-19 pandemic were ripe, including financial concerns, and increases in caregiver stress, mental health difficulties, and substance use (9–11). Indeed, at the beginning of the pandemic, experts sounded the alarm of the “pandemic paradox” (12), where public health measures enacted to keep children medically safe and to control the spread of the virus, such as school closures and stay-at-home orders, may have inadvertently increased exposure to family violence and child maltreatment. Increases in child maltreatment may have implications for children and adolescents' mental health in both the short and long term. In this narrative review, we provided a brief overview of the detrimental impacts of child maltreatment on child and adolescent mental health. We present the evidence for increases in child maltreatment during the COVID-19 pandemic and its spillover effects on children and adolescents' mental health. Using an adapted heuristic model of child maltreatment (13) presented in Figure 1, we discuss the mechanisms by which increases in child maltreatment occurred, including increases in risk factors during the pandemic. Finally, we present protective factors, both pandemic-specific and general, that have the potential to mitigate increases in child maltreatment as well as those that interrupt the negative impact of maltreatment on children and adolescents' mental health. We conclude with key learnings with regard to policies, practices, and directions for future research as they relate to child maltreatment and children's mental health.

### 2.1 The impact of child maltreatment on child and adolescent mental health

Child maltreatment is a well-established risk factor for mental health difficulties in children and adolescents, with 21%–41% of common mental health conditions being attributable to adversities such as maltreatment in childhood (14). Child maltreatment is broadly defined as any form of abuse or neglect directed toward a child under the age of 18 years (15). Maltreatment comes in many different forms, with the most prevalent types being exposure to intimate partner violence, physical abuse, sexual abuse, emotional abuse, and neglect. Neglect is the failure to provide basic needs, such as food, shelter, education, and medical or psychological care (16). Although child maltreatment is often perpetrated by parents or caregivers, it can be perpetrated by any individual in a position of trust or power (15), including teachers, coaches, acquaintances or even strangers. Experiencing maltreatment can pose direct threats to a child's health, development, and mental well-being, having cascading effects on their mental health across the life course.

Despite existing challenges related to underreporting and identifying child maltreatment (15), several studies have documented its short- and long-term impact on children's mental health. Indeed, the effects of maltreatment on children have been observed across behavioural, emotional, and cognitive functioning (17). As discussed in Jaffee's (18) review of the relationship between child maltreatment and psychopathology, children who have experienced maltreatment are at a greater risk of presenting externalizing problems, such as attention-deficit/hyperactivity disorder (ADHD), conduct disorder (CD), and oppositional defiant disorder (ODD) (18). They are also more prone to exhibiting delinquent and antisocial behaviour (18). Similarly, maltreated children face an elevated risk of internalizing symptoms and problems, such as major depressive disorder, anxiety disorders, and posttraumatic stress disorder (PTSD) (18). Interestingly, the risk of psychological disorders following exposure to childhood maltreatment does not appear to vary based on sex, race, or ethnicity (18), pointing to its universally detrimental effects. Overall, child maltreatment has adverse effects on the mental health of children and adolescents. However, to fully grasp the impact of maltreatment, it is essential to consider not only situations involving maltreatment but also instances where multiple types of adversity coexist.

Exposures cluster and co-occur (19, 20). That is, exposure to one form of maltreatment conveys risk for exposure to other adversities (21). The dimensional model of childhood adversity proposes that various types of adversity, including maltreatment, share common underlying mechanisms that shape diverse aspects of neural development (22, 23). Rather than examining the effect of each adversity type in isolation, this theory recognizes the interconnectedness of these experiences and seeks to identify common threads that contribute to their impact on neural development while understanding that different types of adversity have different degrees of harm. The dimensional model delineates two underlying mechanisms influencing neural development: threat and deprivation. Threat exposures involve situations that pose a risk of physical harm to a child, such as exposure to intimate partner violence, sexual abuse, or physical abuse. By contrast, deprivation exposures signify a lack of environmental experiences, such as neglect (22). Each of these experiences—whether characterized by threat or deprivation—results in distinct neural and, consequently, developmental outcomes (22). Specifically, deprivation has been linked to an increased risk of externalizing problems, whereas threat has shown longitudinal associations with internalizing and externalizing problems (24). Therefore, considering the type and cumulative nature of maltreatment exposures is critical for understanding its impact on children and adolescents' mental health.

The type of adversity that children and adolescents face is not the only factor that influences developmental and mental health outcomes; timing also matters. According to the sensitive period model, the time at which a child is exposed to adversity is a critical factor in determining its impact on psychopathology symptom outcomes (25). This model suggests that the impact of maltreatment is more likely to be pronounced when it happens during specific periods marked by peak brain plasticity, such as the early years (26). In a study conducted by Dunn et al. (26), results found that psychopathology symptom scores were highest for girls exposed to harsh physical discipline at age nine years and for boys exposed at age five years, indicating that these ages represent peak developmental periods. McLaughlin et al. (27) also suggest that exposure to violence, such as physical abuse, during times when neural systems are highly adaptable to learning about threat and safety can lead to changes in these circuits to rapidly identify threats in their environment. Taken together, developmental age and timing of exposure to maltreatment play critical roles in the development and manifestation of mental health difficulties following maltreatment.

#### 2.1.1 The impact of child maltreatment on child and adolescent mental health during the COVID-19 pandemic

To date, very limited research has directly investigated the impact of maltreatment on mental health in the context of the COVID-19 pandemic. One study conducted in Germany, using an adult sample, found that pandemic-related stressors fully mediated the relationship between past experiences of child maltreatment and mental health outcomes, such as anxiety, stress, and psychological well-being (28). Findings demonstrate an association between past trauma and increased risk of mental health difficulties during the pandemic. Moreover, a study by Salmon et al. (29) found that 16- to 21-year-olds who had a history of adverse childhood experiences were more likely to report heightened levels of stressors and symptoms related to the pandemic. Despite these studies relying on adult samples, such results may be extrapolated to children and adolescents.

To our knowledge, only two studies have been conducted to examine the impact of maltreatment on child and adolescent mental health in the context of the COVID-19 pandemic. In a cross-sectional Chinese study conducted by Bai et al. (30), children who experienced maltreatment during the pandemic and were exposed to or infected with COVID-19 exhibited heightened levels of mental health symptoms, such as aggressive behaviour, depression, and social problems. The study sample consisted of 1,286 children aged 0–10. Moreover, Liu et al. (31) conducted a cross-sectional study in the United States and the District of Columbia. They found a direct association between exposure to child maltreatment during the pandemic and increased mental health problems and suicidal ideation, pointing to the consequential effects of child maltreatment on mental health.

Despite the limited evidence examining the impact of maltreatment on the mental health of children and adolescents during the pandemic, decades of research in developmental psychopathology suggest that the risk factors present during the pandemic would have exacerbated this association. In the following paragraphs, we provide evidence for an increase in child maltreatment during the pandemic and, using a heuristic model, discuss mechanisms for the impact on child and adolescent mental health.

### 2.2 Increases in child maltreatment during the COVID-19 pandemic

Clinicians and researchers internationally warned of an increase in child maltreatment during the COVID-19 pandemic [e.g., (32)], especially for children with pre-existing risk factors such as low household income, caregiver stress, and caregiver mental health difficulties [e.g., (33)].

Although risk factors for child maltreatment were present and, in some instances, intensified during the pandemic, the literature presents conflicting evidence regarding how the pandemic affected rates of child maltreatment. A systematic review by Huang et al. (34) examined studies that assessed the prevalence and changes related to child maltreatment before and during the pandemic (between January 2020 and August 2022). Eight studies found a decrease in the prevalence of child maltreatment during the pandemic, and thirteen studies found an increase. This discrepancy raises questions about the mechanisms influencing the prevalence of child maltreatment during the pandemic, as well as how differences in measurement and context may be impacting results.

One possible reason for discrepancies across studies in the meta-analysis conducted by Huang et al. (34) is the sources of data that were used. Many of the studies that reported a reduction in child maltreatment during the pandemic relied on administrative data collected by governmental organizations, such as child protective agencies and police reports (35). For example, in the United States, Whelan et al. (36) found a 25% decrease in criminal charges associated with child maltreatment. Moreover, Musser et al. (37) found a significant reduction in the number of children entering the foster care system. Similar results were found in Canada; Katz et al. (38) found a 30%–40% decrease in child abuse reports. Taken at face value, these studies indicate that during the pandemic, rates of child maltreatment decreased. However, it is crucial to consider that most administrative data heavily depend on professionals reporting instances of child maltreatment and thus on children and adolescents having direct contact with those who are mandated to report. As a result of stay-at-home orders and the shift to virtual learning, the contact children and adolescents had with mandatory reporters was significantly restricted, especially given that educational staff are primary reporters of child maltreatment (39). After the shift to virtual learning, there was a 58% decrease in child maltreatment reports compared to previous years (40). Such a decrease is thought to be attributed to a reduction in reports and not necessarily to a decline in child maltreatment overall. The limited contact between children and mandatory reporters may make administrative child maltreatment data unreliable to accurately assess the pandemic's impact on child maltreatment.

As it is possible that administrative child maltreatment data were compromised due to the lack of contact between children and adolescents and mandatory reporters, alternative sources of data must be considered. Studies within Huang et al's (34) systematic review that indicated an increase in child maltreatment predominantly utilized data collected through individual surveys, professional evaluations, and unconventional sources such as social media postings and helpline calls (35). In studies examining caregiver questionnaires, there was overwhelming evidence suggesting increased maltreatment in households during the pandemic (41–43). Moreover, studies that utilized data reported directly by child protection teams and social workers to evaluate and confirm instances of child maltreatment found increases during the pandemic compared to pre-pandemic (44, 45).

Examining unconventional data sources, such as social media postings, also provides insight into the prevalence of maltreatment during the pandemic. During the pandemic, Reddit saw an alarming 94% increase in posts related to children and adolescents experiencing maltreatment (46). Further, since March 2020, the Rape, Abuse, and Incest National Network (47) in the United States has reported a surge of calls from minors experiencing maltreatment. Moreover, during the pandemic, there was an increased number of calls and reports to poison control units, suggesting a decrease in caregiver supervision (48). Such sources of data provide evidence that child maltreatment increased during the pandemic and do so by relying on data that is not confounded by the need for child contact with mandatory reporters. As such, there is growing evidence that maltreatment likely increased during the pandemic. In the following sections, we presented a heuristic model of the risk factors that may have exacerbated maltreatment and subsequent child mental health difficulties during the COVID-19 pandemic (Figure 1). We also discuss resilience processes wherein protective factors may have mitigated these adverse outcomes.

### 2.3 How the COVID-19 pandemic exacerbated the impact of child maltreatment on child and adolescent mental health

The heuristic model presented in Figure 1 shows a direct effect of child maltreatment on child and adolescent mental health difficulties, as well as mediator variables that operate as potential mechanisms to explain this association. In a scoping review by Afifi et al. (49), characteristics such as emotion regulation, social relationships, hypervigilance, and reward responsiveness were identified as mechanisms by which child maltreatment exerts its influence on children's mental health. Overall, the proposed conceptual figure captures the multifaceted dynamics among COVID-19 impacts, predisposing factors, COVID-19 risk factors, protective factors for child maltreatment, child maltreatment, mechanisms by which maltreatment affects mental health, protective factors for mental health, and lastly, child and adolescent mental health. This comprehensive framework contributes to a nuanced understanding of the complexities involved in assessing and addressing the impact of the COVID-19 pandemic on child maltreatment and mental health.

### 2.4 Mechanisms for increases in child maltreatment during the COVID-19 pandemic

To understand the increase in prevalence or rates of child maltreatment during the pandemic, it is crucial to adopt a comprehensive perspective. Such a perspective involves assessing both direct and indirect risk factors for maltreatment and understanding how the pandemic increased these risks. Developmental psychopathology theory defines risk as any individual or environmental factor associated with the increased likelihood of developing negative or undesirable outcomes (50). In this context, we examined various risk factors (as depicted on the far-left side of Figure 1) that are likely to have influenced the occurrence of child maltreatment during the COVID-19 pandemic. Broadly, risk factors for child maltreatment that increased during the COVID-19 pandemic include significant disruptions in access to support services, decreased social support, loss of employment, financial hardship, and various predisposing factors, which subsequently led to increases in caregivers' stress, mental health issues, and substance use difficulties.

#### 2.4.1 COVID-19 related impacts

Initially, pandemic-related policies and restrictions resulted in the closure of many essential social services, such as childcare centres, provincially funded parenting programs, and specialized clinical support services. These measures were implemented to limit the virus's spread and reduce infection. However, according to the family stress theory, the reduction or complete absence of access to key external social support may have aggravated pre-existing vulnerabilities among caregivers (51), consequently increasing the risk of child maltreatment. Similarly, during COVID-19 lockdowns, parents and caregivers faced increased challenges in receiving instrumental support from friends and family. Caregivers perceiving a diminished emotional or social support system may have been more prone to child abuse (52). For children, the inability to attend school or daycare meant they lacked supervision and were cut off from a primary source of basic necessities (40), posing a specific risk factor for neglect. Furthermore, they no longer had regular interactions with educational personnel who play a vital role in reporting child maltreatment (40).

Relatedly, pandemic-induced social isolation may have contributed to an elevated risk of child abuse (53, 54), as social isolation was found to be directly associated with caregivers’ verbal aggression, physical punishment, and neglectful behaviour toward their children (55). Feelings of loneliness and worry (43, 54), along with weakened social networks that typically buffer harsh parenting practices (54), may explain the heightened prevalence of child maltreatment in this context.

Another major disruption in parents' and caregivers' lives introduced by the COVID-19 pandemic was unemployment and financial losses. Predictably, both job losses and the resultant financial setbacks (56–58), as well as instances of unemployment (54, 59) are positively correlated with child maltreatment. Economic hardship, which could have manifested itself through mass job cuts during the pandemic, is a well-recognized risk factor for child neglect (59, 60). Similarly, caregiver economic pressure is positively associated with the potential for child abuse and adverse child mental health outcomes (35, 41, 52, 53). An additional study found that housing instability was associated with an increased number of self-reported maternal maltreatment behaviours, while food insecurity was associated with increased use of mother-to-child physical and psychological aggression (61). Moreover, the death of a parent is found to be associated with increased risk of parental and financial stress, and child maltreatment (62). These financial circumstances were widespread, and their combination was even more detrimental to families, distinctly impacting the incidence of child maltreatment.

#### 2.4.2 Predisposing factors

The COVID-19 pandemic created numerous additional challenges for parents and families, including loss of income, reduced social support, and confinement, all of which were found to be associated with risk factors for child maltreatment during the pandemic. In addition to the COVID-19-related impacts, various predisposing factors were also found to be associated with risk factors for child maltreatment during the pandemic. The likelihood of experiencing violence and abuse during the pandemic increased significantly for groups that were already vulnerable prior to the pandemic. Specifically, children living in families that were facing financial hardships, where parents had challenges with mental illness or substance use, and those in one-parent households prior to the pandemic were at increased risk of child maltreatment during the pandemic (63). Moreover, children from families with low perceived affluence and those reporting parental issues like incarceration had a notably higher risk of experiencing maltreatment, both before and during the pandemic (64). Furthermore, Buffarini et al. (65) found that mothers who reported five or more adverse childhood experiences (ACEs) were at a significantly higher risk of reporting having maltreated their children during the pandemic than mothers without ACEs (65), suggesting intergenerational trauma was a risk factor for maltreatment during the pandemic.

In addition to past parental experiences, factors that may predispose a child to the risk of child maltreatment must be considered as well. Augusti et al. (63) conducted a study to examine risk factors for abuse during the pandemic, and pre-pandemic victimization was found to be the most accurate predictor of abuse during lockdown. Moreover, Guo et al. (66) found that children and adolescents who experienced maltreatment pre-pandemic were at increased risk of COVID-19 negatively affecting their mental health compared to peers who were not maltreatment prior to the pandemic.

Children with special education needs saw a significant increase in physical abuse during the pandemic. According to Tso et al. (67), during the pandemic, 25.5% of children with special educational needs experienced at least one episode of severe physical abuse, and 1.9% experienced at least one episode of very severe physical abuse (67). Furthermore, they found that children who were diagnosed with mental disorders prior to the onset of the pandemic were at a heightened risk of experiencing severe physical abuse compared to those without a diagnosed mental disorder (67). Overall, these findings emphasize how existing vulnerabilities, especially those tied to socioeconomic status, past trauma, and biological factors, heighten the risk of child maltreatment during the pandemic.

#### 2.4.3 COVID-19 risk factors for child maltreatment

Continuing the challenges described previously, the pandemic era saw a notable decrease in caregivers' mental health and overall well-being. Specifically, caregivers experienced a surge in stress levels as they grappled with virus transmission-related fears, navigated unexpected job losses and shifts, adapted to newly imposed policies and restrictions, worked remotely, and took on additional responsibilities like homeschooling (11). Since the onset of the pandemic, caregivers have reported a deterioration in their mental health, reduced patience toward their children, and greater feelings of being overwhelmed by the demands of parenthood (68). These findings are particularly concerning, given that caregiver stress constitutes a major risk factor for child maltreatment (69). Amidst pandemic-related fears and a constant state of uncertainty, there is an increased perception of risk, which has the potential to further deteriorate caregivers' mental health (41, 53). Naturally, these circumstances may create obstacles for caregivers in effectively addressing their children's needs, paving the way for neglect and other forms of maltreatment (70).

Other adverse mental health outcomes in caregivers amplified by the COVID-19 pandemic include anxiety and depression, both of which are positively linked to the potential for child abuse (35, 41, 52). Furthermore, worries, feelings of loneliness, family stress and dysfunction (11, 52, 53, 71), along with caregiver substance use (63), each pose a risk of increasing child maltreatment. Other forms of family violence, such as exposure to intimate partner violence or intimate partner violence in the home, also contributed to an elevated risk of child abuse (33). It should be mentioned that in addition to caregivers’ mental health struggles, financial difficulties, and heightened social stressors being risk factors for child maltreatment during and since the COVID-19 pandemic, they were also found to be risk factors for decreases in child mental health during the pandemic (72). In essence, the combined factors of caregivers' mental health struggles, financial difficulties, and heightened social stressors underscore the multifaceted and complex nature of increased risk factors for child maltreatment during and since the COVID-19 pandemic.

### 2.5 Resilience in the face of child maltreatment during the COVID-19 pandemic

Despite the pernicious effects of maltreatment on the mental health of children and adolescents, not all children were exposed to maltreatment during the pandemic, and not all children exposed to maltreatment developed mental health concerns. In fact, previous research has shown that between 12% and 22% of maltreated children demonstrate competence across multiple domains of functioning over time (18) and 35% of children exposed to maltreatment are never diagnosed with a mental health disorder (73). Furthermore, some individuals experience posttraumatic growth in the aftermath of maltreatment. Posttraumatic growth refers to positive psychological changes leading to an increased sense of meaning or benefit from the negative experience (74). For example, a study by Wright et al. (75) found that 87% of women who had experienced child sexual abuse reported some growth in the aftermath of their experience, including personal growth, growth in relationships, or spiritual growth. Overall, it is critical to understand the processes and mechanisms by which adaptive mental health can be attained for children exposed to maltreatment.

Resilience science offers a framework to consider the processes by which children exposed to maltreatment during the COVID-19 pandemic may have experienced better-than-expected mental health outcomes. From a systems perspective, resilience is the capacity and processes by which a system adapts successfully despite challenging circumstances (76). Resilience refers to the processes and mechanisms by which adaptive outcomes occur rather than an individual trait or a simple outcome. Thus, when considering the impact of maltreatment on the mental health of children and adolescents during the COVID-19 pandemic, resilience processes would be those that promote good mental health and protect against the toxic effects of maltreatment.

Decades of research in resilience science have focused on the promotive and protective factors that lead to adaptation in the face of maltreatment. Promotive factors are those that have a direct positive effect on the outcome (i.e., a main effect) (77). For example, a supportive caregiver-child relationship has been shown to be associated with lower levels of internalizing symptoms among children exposed to maltreatment (78). Protective factors are those that mitigate the likelihood of a poor outcome in the face of risk. For example, at a community level, the collective efficacy of a neighbourhood, including its cohesion, has been shown to reduce the association between childhood neglect and adolescents' aggression (79). Both promotive and protective factors can exist at different levels of the social ecology (i.e., individual, familial, community, or societal level) and may differ depending on the child's developmental stage (80, 81). A scoping review conducted by Afifi and MacMillan (49) identified factors across the social ecology that are associated with adaptive mental health in the face of maltreatment, including self-regulation abilities, self-efficacy, supportive family relationships, and social support outside the family. Below, we detail promotive and protective factors (resilience factors) that may have both mitigated the impacts of risk on child maltreatment during the COVID-19 pandemic as well as protective factors for the development of mental health concerns following maltreatment during the pandemic.

### 2.6 Protective factors for child maltreatment exposure during the COVID-19 pandemic

The COVID-19 pandemic brought forth many factors that increased the risk of child maltreatment, yet maltreatment did not increase universally across all families. Many families exhibited protective factors that effectively reduced the risk of child maltreatment (See Figure 1). Higher socioeconomic status was a critical factor in preventing child maltreatment during the pandemic (56, 58, 82). Elevated socioeconomic status served as a protective factor against child maltreatment, given the reduced risk of caregivers experiencing unemployment or stress associated with potential job loss and, as such, financial stress (82). Conditions such as unemployment are strongly linked to subsequent stressors, including economic difficulties, housing instability, and food insecurity (83). These factors, in turn, escalate the risk of child maltreatment by almost 90% (84). The economic strain and job loss triggered by the pandemic thus increased the risk of child maltreatment substantially (84). However, those with higher socioeconomic status were more likely to be shielded from these risk factors. Furthermore, in families with higher socioeconomic status, the transition to remote work emerged as an additional protective factor against child maltreatment (85, 86). Remote work was found to contribute to caregiver well-being by offering autonomy and schedule flexibility, thus acting as buffers against caregiver distress and negative parenting behaviours.

In addition to socioeconomic factors, maintaining caregiver well-being emerged as a crucial protective factor in reducing the risk of child maltreatment during the pandemic (52, 87). As a result, protective factors for caregiver mental health are indirectly protective factors against child maltreatment. Factors such as caregivers developing and utilizing coping strategies to manage stress and access to mental health services have been identified as strong protective factors against caregiver mental health decline (34, 88). Further, caregiver social support has been identified as a protective factor against both caregiver mental health decline and child maltreatment during the pandemic (52). Research indicates that help and support from others can enhance a caregiver's capacity to manage stressors and improve their mental and physical health (89).

Moreover, certain family practices supported caregiver well-being during the pandemic. A study by Rosen et al. (90) emphasized the importance of structured routines, limited passive screen time in the home, and lower exposure to pandemic-related news in protecting against caregiver mental health decline. When considered together, such studies highlight the need to support caregiver mental health to decrease the risk of child maltreatment (41). Although there is little research on caregiver qualities as protective factors against child maltreatment, empathy has been identified as an important caregiver quality. Yamaoka et al. (91) reported that high caregiver empathy was a protective factor against child maltreatment during the pandemic.

Amid the challenges of the COVID-19 pandemic, various protective factors against child maltreatment were present. Such factors included familial, social, and individual dimensions, thus highlighting the importance of secure employment and caregiver mental health in mitigating the risk of child maltreatment during challenging times.

### 2.7 Protective factors for child and adolescent mental health following maltreatment during the COVID-19 pandemic

There is mounting evidence that child maltreatment during the COVID-19 pandemic was associated with an increase in mental health problems in children (30). Indeed, child maltreatment was the strongest predictor of mental health problems in children under the age of 10 after accounting for caregiver conflict, COVID-19 exposure, and discipline (30). Although studies identified the direct association between exposure to child maltreatment during the pandemic and increased mental health problems and suicidal ideation (31), limited research has examined factors protecting against the development of mental health challenges for children exposed to heightened maltreatment during the pandemic.

A vast literature on child trauma and developmental psychopathology has documented the factors that could have prevented the development of mental health difficulties among children and adolescents following maltreatment during the pandemic. Specifically, a systematic review by Meng et al. (92) details factors associated with more optimal mental health following exposure to child maltreatment, such as individual coping skills, positive caregiver-child relationships, and community social support. For example, positive caregiver behaviours can mitigate the development of psychopathology in children who were exposed to physical abuse (93). Social support outside the family home from individuals such as teachers, coaches, or peers has also been consistently identified as mitigating the risk posed by maltreatment to mental health (94). That is, relationships that provide emotional and instrumental support can help to reduce stress and increase coping skills, which leads to positive mental health outcomes in the face of adversity.

Although there is strong evidence related to these protective factors prior to the pandemic, it is important to consider how the social and environmental context of the pandemic may have adversely impacted protective factors that were previously established. That is, the caregiver-child relationship may have been compromised due to elevated levels of caregiver mental health difficulties, stress, substance use, and family violence (9, 11, 95). Thus, the caregiver-child relationship may not have been able to function as adequately due to these stressors. Indeed, hostile caregiver behaviour during the COVID-19 pandemic was shown to contribute to elevations in internalizing and externalizing difficulties among children between 7 and 9 years of age (96). Similarly, access to community supports were significantly reduced during the COVID-19 pandemic due to restrictions on social gatherings, cancellation of extracurricular activities, and social distancing requirements. As such, simple access to individuals and relationships that would have otherwise served as protective were restricted, leading to a decreased likelihood of their protective role. Although limited evidence currently exists on protective factors for the development of mental health difficulties following maltreatment during the COVID-19 pandemic, we hypothesize that many protective factors that would typically be at play were significantly disrupted, potentially leading to an increase in mental health difficulties among children exposed to maltreatment.

### 2.8 Implications for policy, practice, and research

There are several important take-aways from this review on child maltreatment and children's mental health during the COVID-19 pandemic. In the following section, we summarize the key findings from our narrative synthesis and discuss implications for policy, practice, and future research.

#### 2.8.1 Implications for policy

Examining diverse sources of evidence on the prevalence of maltreatment during the pandemic, it is highly likely, especially among families experiencing high levels of stress and income insecurity, that maltreatment increased during the COVID-19 pandemic. Additionally, an increase in risk factors for child maltreatment, including intimate partner violence, caregiver mental health difficulties, lack of social support, and socioeconomic challenges, are very clearly documented. Indeed, a paradox emerged whereby public health practices that were meant to keep children medically safe put families at increased risk for violence and maltreatment. Given that the pandemic led to an increase in challenges for caregivers, caregiver and family well-being must be at the forefront of pandemic-related preparedness and decision-making to reduce maltreatment and its impacts. The main sources of stress for caregivers during the pandemic that are associated with maltreatment include lack of childcare, income loss, and loss of support from social networks (97). As such, policies that prioritize continuity of childcare and school attendance for children may be critical for reducing caregiver stress and mental health challenges. Furthermore, such policies keep children visible, a characteristic that is critical for child maltreatment prevention. Additionally, policies that maintain employment as well as financial support for families in the face of job loss are also key to reducing caregiver stress.

Preliminary evidence suggests that child maltreatment during the pandemic was associated with mental health difficulties in children (30). Therefore, policies that prioritize child and adolescent mental well-being, such as attending school, extracurricular activities, and social programs, are critical to reducing the negative impacts of maltreatment. Schools and community mental health services are also one of the primary sources of child maltreatment reporting, which decreased substantially during the COVID-19 pandemic (98) despite an increase in the severity of abuse presentations in hospitals (99, 100). This is why the American Academy of Pediatrics and the Royal Society of Canada issued statements during the pandemic urging children's safe return to school to mitigate poor outcomes (101, 102). Schools and community programs provide instrumental protection and support to the mental health of children and should be prioritized to be kept open in future.

#### 2.8.2 Implications for practice

Many children's mental health services that provide psychosocial and therapeutic support to children who have been maltreated pivoted to online service delivery during the pandemic. Although online service delivery maintained some access to therapeutic supports, this approach has many limitations, particularly for children and adolescents who may not be living in safe contexts (103). Specifically, not all children and adolescents have access to a safe space to speak to a service provider, and confidentiality may be limited inside the home. It may also be more challenging for a therapist to assess risk for the well-being of a child without seeing the family in person. As such, prioritizing in-person service provisions, especially for children at the highest risk for maltreatment, are paramount.

Within the child welfare setting, child welfare workers had to significantly alter their service provision during the COVID-19 pandemic (38, 104). For example, in-person contact with families was limited due to public health measures, visiting and meeting with families became more challenging, and case workers experienced very high levels of burnout (105). Literature detailing recommendations for changes in child welfare practices are provided in detail elsewhere (106). Briefly, child welfare workers should be designated as essential workers during the pandemic to maintain timely in-person investigations as well as the management of cases. Additionally, community partnerships with organizations that provide services to children and adolescents should be bolstered to increase opportunities for child welfare reporting as well as provide supports and services to families in need.

Professionals and community organizations must continue providing in-person services during lockdowns or stay-at-home orders. Additionally, they need to be made aware of the potential increase in maltreatment during such times. There is a crucial need for staff to understand how stressful events, like the pandemic, are likely to increase the prevalence of child maltreatment. Further, they need training to recognize and respond to signs and symptoms of child maltreatment. The Violence, Evidence, Guidance, Action (VEGA) project (107) is an evidence-based educational tool designed to aid healthcare and social service providers to safely identify and respond to family violence. The implementation of this tool could enhance professionals' capability to identify child maltreatment, potentially leading to improved child safety.

#### 2.8.3 Implications for research

A glaring gap in the literature is high-quality, population-level data on whether maltreatment increased during the COVID-19 pandemic. Previous research has pointed to the importance of rigorous national prevalence studies of child maltreatment to inform prevention and intervention efforts (108). The inability to have a clear understanding of whether maltreatment increased or not at a national level is a surveillance failure. Furthermore, it can often be challenging for researchers to ask about child maltreatment in research surveys due to difficulties with approvals from Institutional Review Boards (109). Specifically, concerns are often raised regarding distress induced to participants, a duty of care to follow up with participants, and legal liabilities. However, surveys on the prevalence of maltreatment, particularly during public health or environmental emergencies, are necessary to inform prevention and social policies. It is especially important for these surveys to be routine to be able to chart changes and trends over time, as well as document differences before and after significant events.

Another important research gap pertains to research studies examining mechanisms that explain “why,” “how,” and “under what circumstances” maltreatment and subsequent mental health difficulties occurred during the COVID-19 pandemic. Thus, in addition to collecting ongoing nationally representative longitudinal prevalence data on child maltreatment, it is also important to collect information on key protective factors and related health outcomes that may be candidates for interrupting poor outcomes. Finally, as mentioned previously, there is a need for investment in continual longitudinal surveillance of maltreatment at a national level to inform key decision making.

## 3 Conclusion

For children and families who were at the highest levels of risk for maltreatment during the pandemic, the current review paints a stark picture. There is clear evidence for increased risk factors associated with child maltreatment, as well as burgeoning evidence that child maltreatment also increased during the pandemic. In addition to increased exposure to maltreatment, protective factors for both child maltreatment and the development of mental health difficulties following maltreatment, such as positive caregiver-child relationships, social support, and peer relationships, were severely disrupted during the pandemic. Furthermore, there was limited surveillance and reduced access to community services and supports that would typically intervene and provide supports to families experiencing maltreatment. Taken together, the current review provides strong justification for changes in policies and practices as they relate to children and families at risk of maltreatment during future public health and environmental emergencies. Ensuring that the needs of children exposed to maltreatment, arguably some of the most vulnerable children in society, are protected and supported will be consequential in ensuring their optimal mental health.
